# Upper Limb Position Tracking with a Single Inertial Sensor Using Dead Reckoning Method with Drift Correction Techniques

**DOI:** 10.3390/s23010360

**Published:** 2022-12-29

**Authors:** Lu Bai, Matthew G. Pepper, Zhibao Wang, Maurice D. Mulvenna, Raymond R. Bond, Dewar Finlay, Huiru Zheng

**Affiliations:** 1 School of Computing, Ulster University, Belfast BT15 1ED, UK; 2 School of Engineering, University of Kent, Canterbury CT2 7NZ, UK; 3 Department of Medical Physics, East Kent Hospitals University NHS Foundation Trust, Canterbury CT1 3NG, UK; 4 School of Computer and Information Technology, Northeast Petroleum University, Daqing 163318, China; 5 School of Engineering, Ulster University, Belfast BT15 1ED, UK

**Keywords:** upper limb motion monitoring, inertial sensor, zero velocity update, dead reckoning, wavelet analysis, high-pass filter

## Abstract

Inertial sensors are widely used in human motion monitoring. Orientation and position are the two most widely used measurements for motion monitoring. Tracking with the use of multiple inertial sensors is based on kinematic modelling which achieves a good level of accuracy when biomechanical constraints are applied. More recently, there is growing interest in tracking motion with a single inertial sensor to simplify the measurement system. The dead reckoning method is commonly used for estimating position from inertial sensors. However, significant errors are generated after applying the dead reckoning method because of the presence of sensor offsets and drift. These errors limit the feasibility of monitoring upper limb motion via a single inertial sensing system. In this paper, error correction methods are evaluated to investigate the feasibility of using a single sensor to track the movement of one upper limb segment. These include zero velocity update, wavelet analysis and high-pass filtering. The experiments were carried out using the nine-hole peg test. The results show that zero velocity update is the most effective method to correct the drift from the dead reckoning-based position tracking. If this method is used, then the use of a single inertial sensor to track the movement of a single limb segment is feasible.

## 1. Introduction

Neurological disorders are the leading cause of disability-adjusted life years (the sum of years of life lost and years lived with disability) and the second leading cause of death according to recent research on the global burden of neurological disorders [1]. The report on neurological prevalence published by the UK Neurological Alliance, states that there are an estimated 14.7 million neurological cases in the UK. This equates to at least 1 in 6 people living with one or more neurological conditions [2]. Severe neurological conditions such as stroke, Parkinson’s disease, traumatic brain injury, spinal cord injury, motor neuron disease and multiple sclerosis can result in the impairment of limb mobility, and difficulties in carrying out normal cognitive tasks (learning and communication) [3].

Between 73% and 88% of first-time strokes result in an acute hemiparesis of the upper and/or lower limbs [4]. A significant number of patients who had a stroke had one of their arms affected [5]. A survey of stroke patients with arm and leg paresis showed that more than 75% of the patients on admission to a stroke unit required significant assistance with upper limb function recovery [6]. Rehabilitation programs involve occupational therapy and physiotherapy to help the patients to ease the symptoms, regain upper limb mobility and, furthermore, lead an independent life. Our previous work [7] used low-cost gaming sensors to assess the upper limb movement of patients pre and post-Botulinum Toxin treatment. Additionally, remote rehabilitation using automatic recognition of physical exercise is getting more attention [8]. With the use of the deep-learning-based method, Bijalwan et al. [9] proposed a model for post-injury walking pattern restoration and postural stability rehabilitation exercise recognition. In addition, there are also speech and language therapists, nurses and other specialists working together in a typical multidisciplinary rehabilitation team. In particular, it has been shown that in terms of regaining independence in everyday living, improving upper limb function is one of the important factors in a patient’s rehabilitation [10,11,12], and research indicates that more attention is needed for the treatment of upper extremity impairment [13,14,15]. Therefore, having an effective rehabilitation program and an objective means of monitoring the efficacy of that program should be of value to both clinicians and patients.

Typical tests to monitor response to rehabilitation include the nine-hole peg test, drinking test and bean bag test [16]. Currently, data from these tests are usually restricted to the use of a stopwatch to measure test completion time and visual observation of the limb trajectory. A more objective measure would provide additional information to the clinician. One means to do this is to use inertial sensors to monitor limb segment motion. The use of inertial sensors can provide information on additional parameters such as the timing of specific movements within the test, acceleration, velocity profiles, movement smoothness and the path followed by the limb during the tests.

The development of Micro Electro Mechanical Systems (MEMS) technology has resulted in the availability of small inertial sensors which are designed to be attached to the upper limb of a person. In recent years, inertial sensors are commonly used to combine data from accelerometers, magnetometers and gyroscopes to measure the change in position and orientation [16,17,18,19,20]. Initially typical applications were focused on tracking head motion where the accelerometer was used as an inclinometer and the gyroscope used to sense changes in orientation [21,22]. However, it was realised that in order to improve the accuracy of measurement, data from magnetometers had to be fused with that from the accelerometers and gyroscopes [23,24]. An inertial measurement system will usually consist of several inertial sensors and a biomechanical model to interpret the sensor data. Previous work focused on upper limb motion sensing using multiple sensors and kinematic modelling [18,25,26,27]. There is, however, a growing need to adopt a less complex system to reduce the cost and improve user compliance and ease of use. To date, there has been a lack of studies that use a single inertial sensor for upper limb motion monitoring. Leuenberger et al. [28] proposed a single wrist-worn inertial sensing measurement unit to quantitatively assess the upper limb function of stroke survivors. However, the results are mainly reported for a range of motion measurements, whereas limb trajectory is also of clinical importance.

One approach to monitoring limb trajectory using inertial sensors is the Dead Reckoning method (DR) [29]. The DR method has been used to estimate pedestrian navigation using inertial sensors attached to a person’s lower limbs [30,31]. Labinghisa et al. [32] used empirical mode decomposition to remove the drifts from the pedestrian dead reckoning. Ju et al. [32] proposed an advanced heuristic drift elimination approach which used ZUPT method. Elbes et al. [33] proposed a gyroscope drift correction method in support of pedestrian dead reckoning and their proposed approach was able to remove more than 85% of the drift. Dead reckoning is a navigation technique widely used in inertial tracking for ships and pedestrians. For those applications, errors within one meter can be accepted. However, for the application of human upper limb motion position tracking, the errors should be within one centimetre. To the authors’ best knowledge, there is no research has been conducted in applying dead reckoning in human upper limb monitoring. This method is of interest because, if successful, it would enable simpler setups for some clinical tests using only one sensor to measure the movement of a particular upper limb segment, e.g., the hand. This would be attractive in terms of system cost and set-up time. The accuracy in the position monitoring for upper limb motion is important in understanding patient performance, especially for movement related to hand and wrist dexterity.

To track upper limb segment position with a single inertial sensor, the DR method is mandatory. However, the usefulness of DR method is limited because of the presence of unavoidable system errors. These errors are due to the presence of sensor offsets and offset drift—described as random walk [34,35,36]. The random walk error [37] presents as a low-frequency component in limb segment movement. There are several techniques available to minimise the impact of this low-frequency drift. To compensate the system error, the Zero Velocity Update (ZUPT) [38], has been widely adopted in the area of pedestrian tracking. However, one limitation of this method is the requirement that there are regular intervals where the limb segment is at zero velocity. This is readily achievable when monitoring gait when the forefoot is in contact with the ground [39]. Unlike the gait cycle where zero velocity intervals are present, upper limb segment movements zero velocity events are more difficult to predict. Therefore, in this paper, we evaluate the accuracy of three drift correction methods; the ZUPT [38], and wavelet analysis [40,41,42] [38,39]. and high pass filtering [43].

The remainder of the paper is arranged as follows. In Section 2, the DR method and errors arising from drifts are presented. To reduce the drift, a range of methods including ZUPT, wavelet analysis, high-pass filter are discussed in Section 3 and the results of the drift correction methods are presented in Section 4. Section 5 concludes the paper.

## 2. DR Method

The basis of DR method is to estimate the current sensor position by using the previous position and to double integrate the translational acceleration over elapsed time to estimate the change in position. However, the gravitational component in the measurable acceleration data must first be subtracted from the measurable acceleration by applying the quaternion or rotation matrix data. Additionally, the gravitational component in each axis will change as the sensor orientation changes. Therefore, it is necessary to track the orientation as well as the translational acceleration of the sensor in order to be able to remove the gravitational component from the acceleration.

Therefore, it is challenging to estimate translation based on acceleration measurement alone. Without any correction strategy, the position estimation is only expected to be acceptable for upper limb measurements over a very short time—one or two seconds. For example, a 0.01 m/s^2^ error in acceleration will result in an error in the position of 9 m after 30 s. An error in the estimate of acceleration of 0.1% of the maximum acceleration and will result in a 10% offset error in position after 10 s.

**Figure 1:**
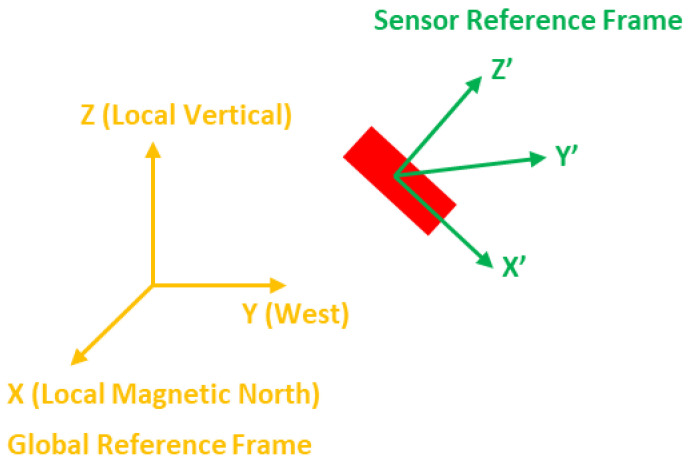
Sensor and global reference frames.

The sensor reference frame is changing all the time with respect to the change in the sensor orientation. In order to obtain the position estimation, a fixed reference frame is needed, and, in this work, global reference frame is used. As shown in Figure 1, the global reference frame is an earth-fixed reference frame in which the positive x direction is pointing to the local magnetic North, the positive y direction is pointing to the West according to the right-handed coordinate system and the positive z direction is pointing up, in the opposite direction to the Earth’s gravitational field.

By using the rotation matrix Rsg(t), the measurable acceleration vector in the sensor reference frame a→s(t) can be converted from the sensor reference frame to the global reference frame to a→g(t). Once the gravitational component $\vec{g}$ has been removed then double integration of the linear acceleration data in the global reference frame a→glinear(t) should, in theory, enable any change in sensor or segment position to be estimated. The equations for calculating linear position [18] are as follows:

(1) a→glinear(t)=Rsg(t)∗a→s(t)−g→

(2) v→glinear(t)=∫ta→glinear(t)dt

(3) p→glinear(t)=∫tv→glinear(t)=∬ta→glinear(t)dt

where a→glinear, v→glinear and p→glinear are the linear acceleration, linear velocity and linear position, respectively, in the global reference frame.

**Figure 2:**
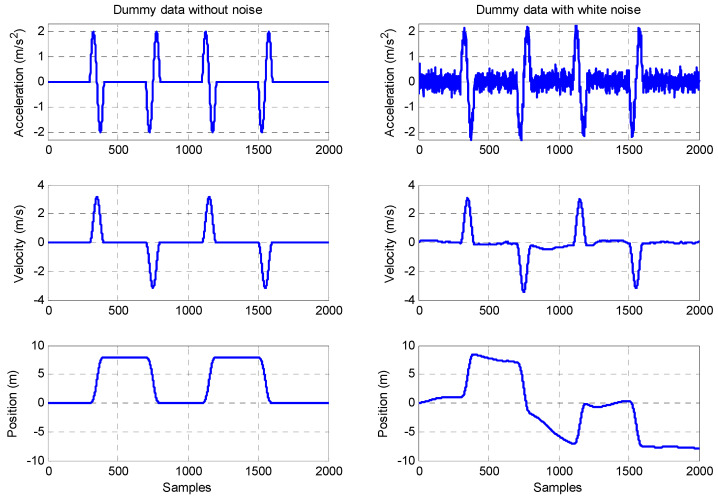
Illustration DR algorithm using dummy data.

Dummy data which represent a typical ideal acceleration profile in one axis are used here to illustrate the DR algorithm for position tracking. In the left column of Figure 1, a sine wave has been used to create the acceleration dummy data whose maximum magnitude is 2 m/s^2^. Velocity and position have been computed by using the DR algorithm, and therefore an ideal example has been made. However, in real measurements, there exists noise and offset in the acceleration measurements. Therefore, the dummy acceleration data with white noise (mean value is 0.2 m/s^2^) were used to evaluate the performance of the DR algorithm when there is noise in the acceleration. The dummy acceleration data with white noise, the corresponding velocity and the position integrated from this acceleration are presented in the right column of Figure 2. It shows the significant error in the tracking of the position when the white noise is included can be up to twice its initial value.

**Figure 3:**
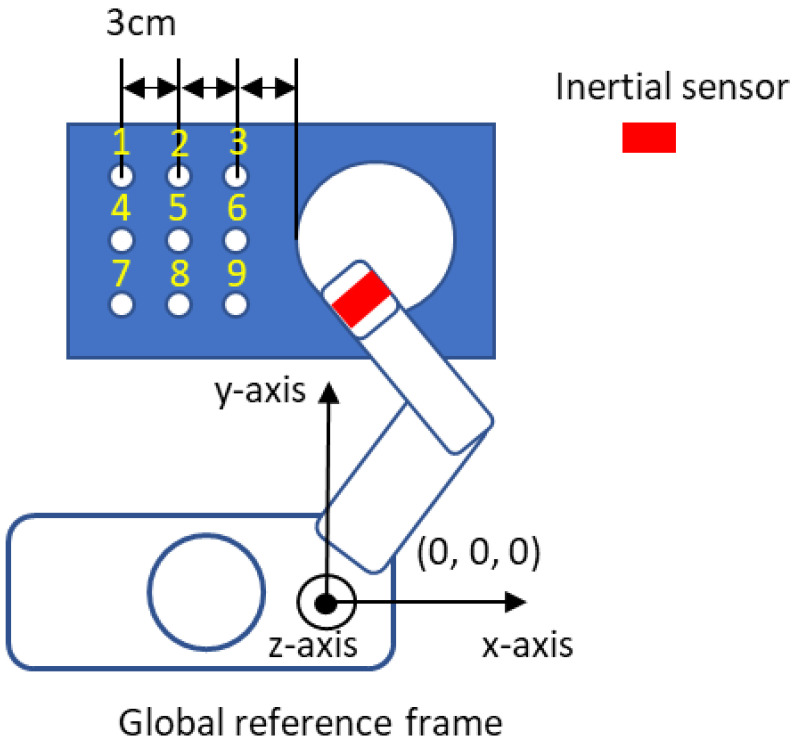
Illustration of NHPT.

**Figure 4:**
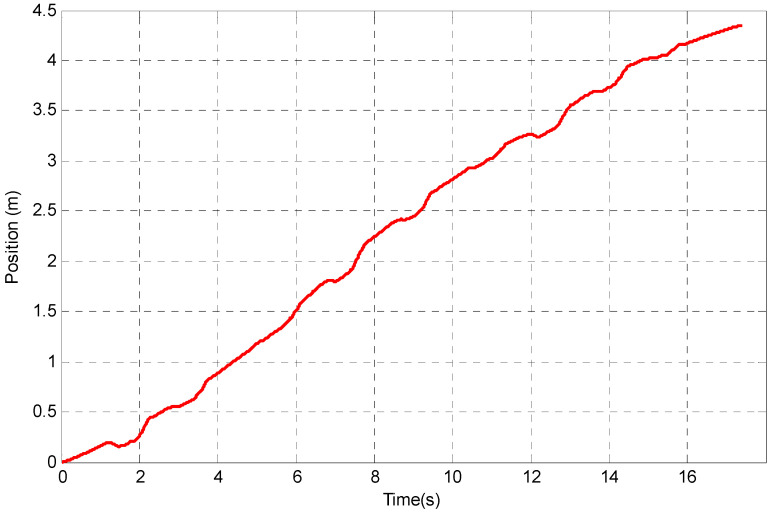
Errors in position tracking using DR algorithm for NHPT.

The evaluation of the DR algorithm uses the dummy data presented, and although this tracking method seems straightforward, it can be seen that when real data are used there will be additional sources of error. These errors arise from uncertainty in the estimate of the gravitational component and offsets and offset drifts inherent in the sensor itself. The presence of these offsets, changes in offsets with time or temperature and noise in the acceleration data can lead to significant errors in the calculation of the velocity and position of the upper limb segments. To demonstrate the use of the algorithm, Nine Hole Peg Test (NHPT) [44] is selected as it is a standard rehabilitation assessment test. The experiment setting of NHPT is illustrated in Figure 3. During the NHPT, the subjects are asked to pick up the pegs from the container one at a time using either their left or right hand and then put the pegs into the holes in any order. To simplify the experiment, the participant is asked to place pegs following the sequence from 1 to 9 as shown in Figure 3. Only one inertial sensor is attached to the hand of the participant by using a Velfoam strapping and is used to track the hand movement. The coordinate system used in the experiment is the global reference frame and the origin of the coordinate selected is the shoulder of the subject. The double integration of any error in the estimation of the offset in the acceleration data can, after a few seconds, result in errors in the calculation of position which are several hundred percent greater than the actual translation of that point as shown in Figure 4—an example of hand position tracking during the NHPT. As can be seen, the errors present when using the DR algorithm can be several orders of magnitude greater than the signals of interest. Even after all the offsets have been removed, a low-frequency error (that can be classified into instrument noise and manipulation errors [45]) may still be present.

This is because the integration or summing of the acceleration signal to calculate velocity will integrate/sum any error in the estimate of offset. This error will increase in direct proportion to the measurement time and sample rate. If the DC offset is not removed from acceleration output or velocity calculation, the effect of errors in the estimation of offset of the position will follow a square law relationship with time and sample rate.

Equation (4) shows how the error in the estimate of velocity is proportional to the acceleration error, offsets and time of measurement and the error in the estimate of the position is proportional to the square of the elapsed time.

(4) $$\{\begin{array}{c} a_{linear}\left(t\right)=a_{linear-ideal}\left(t\right)+w+\theta \left(t\right)\\ v_{linear}\left(t\right)=v_{linear-ideal}\left(t\right)+w\cdot dt+\int \theta \left(t\right)\\ p_{linear}\left(t\right)=p_{linear-ideal}\left(t\right)+1/2\cdot w\cdot {\left(dt\right)}^{2}+\theta \left(t\right) \end{array}$$

In which, the $a_{linear}$, $v_{linear}$ and $p_{linear}$ are the estimated linear acceleration, velocity and position, respectively. dt is the elapsed time. The $a_{linear-ideal}$, $v_{linear-ideal}$ and $p_{linear-ideal}$ are the ideal acceleration, velocity and position without noise. The measured acceleration $a_{linear}$ is composed of the ideal acceleration $a_{linear-ideal}$, an offset (constant value) w from the sensor and an error from the computation of the orientation θ(t). The offset w will cause an error that is proportional to the square of the measurement time. The occurrence of θ(t) is the error in estimating the gravity component that also has to be removed from the total acceleration in order to obtain the linear acceleration. The θ(t) may be the reason that causes a very low-frequency component in the computed position $p_{linear}$.

**Figure 5:**
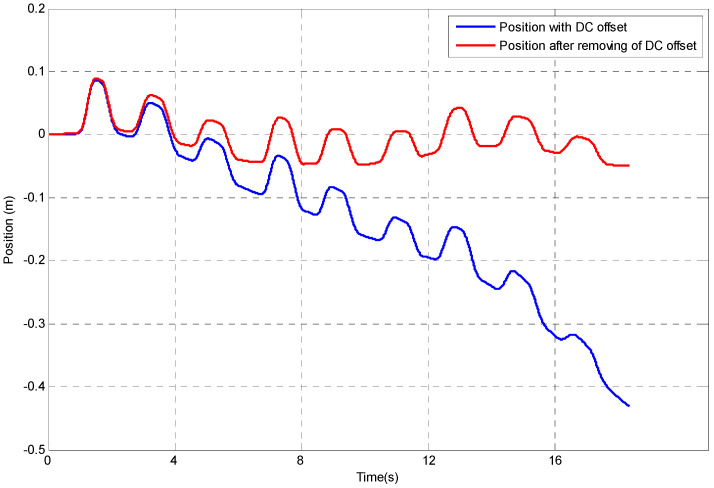
Position tracking before and after DC offset removal.

Therefore, it is challenging to estimate translation based on acceleration measurement alone. Without any correction strategy, the position estimation is only expected to be acceptable for upper limb measurements over a very short time—one or two seconds. For example, a 0.01 m/s^2^ error in acceleration will result in an error in the position of 9 m after 30 s. An error in the estimate of acceleration of 0.1% of the maximum acceleration and will result in a 10% offset error in position after 10 s. As has been seen the errors present when using DR algorithm can be several orders of magnitude greater than the signals of interest, and even after all the DC components have been removed, a low-frequency error (can be classified into instrument noise and manipulation errors [45]) may still be present, as seen in the NHPT data of Figure 5.

There are different noises generated using IMU as it consists of different sensors including accelerometer, gyroscope and magnetometer. For accelerometer, the main noise is DC noise. In this study apart from remove DC offset from the accelerometer, random walk errors appear after double integration, and they still need to be addressed. These errors are due to the presence of sensor offsets and offset drift. As can be seen in Figure 4 and Figure 5, after removing the DC offset, there are still random walk errors from the double integration. The method gives on a possible solution to track the position using a single sensor.

Initial evaluation [46] and evidence from the literature [47] indicate that position estimation is only expected to be acceptable (1% of the total measurement distance) for measurements over a very short time—one or two seconds. Therefore, error correction methods are needed if a single inertial sensor and the DR algorithm are to be used for limb segment position tracking. The dead reckoning position error correction methods ZUPT [48], high-pass filter and wavelet analysis [49], will now be presented.

## 3. Methods

### 3.1. ZUPT

ZUPT is a standard drift correction method which was widely used in inertial navigation systems [50]. This method is based on the assumption that there are times when the segment velocity is known to be zero. For this research, the upper limb evaluation tests for example, NHPT, bean bag test [16] should meet this requirement. At these points in time when the velocity is known to be zero, then the estimated velocity of the segment is reset to zero thus minimising any errors in the velocity estimate accumulated during the previous time period. If it also assumed that the drift in velocity between the rest points is caused by the integration of a constant offset then this drift can be approximated to a straight line of constant slope and a correction for that drift can also be made, thus further reducing the error introduced by the offsets and their changes between the zero velocity points. Doing this should then also reduce the error introduced when integrating the velocity to estimate position. Although the occurrence of zero velocity can be identified by manual examination of the waveforms an automatic method will be required for a practical system. Therefore, in order to automatically estimate the occurrence of zero velocity the double threshold method [51] is used. The short-time signal energy and zero crossing rates (ZCR) (the rate of the signal sign changes) are used to estimate the two thresholds for this double-threshold method. Since gyro data are sensitive to changes in orientation when compared with the accelerometer and magnetometer data, it is chosen as the signal $s=gyro_{y}$ in the equation to obtain the zero velocity intervals. The short-time energy is calculated by:

(5) $$E_{i}=\overset{N}{\underset{n=1}{\sum }}s_{i}{}^{2}\left(n\right)\;\;\;\;\;\;\;\;\;\;\;\;\;\;\;\;\;\;\;\;\;\;\left(s_{i}=gyro_{yi}\right)$$

Additionally, the ZCR is calculated by:

(6) $$Z_{i}=\overset{N}{\underset{n=1}{\sum }}\left|sgn\left[s_{i}\left(n\right)\right]-sgn\left[s_{i}\left(n-1\right)\right]\right|$$

where $sgn\left[s_{i}\left(n\right)\right]=\{\begin{array}{c} 1,s_{i}\left(n\right)\geq 0\\ 1,s_{i}\left(n\right)<0 \end{array}$.

After short-time energy computation, the higher threshold T1 and lower threshold T2 in short-time energy are selected to detect the start and end point to a movement (See Figure 4). The low threshold T2 should be small enough to include every possible velocity greater than zero due to movement and the high threshold should be large enough to exclude every possible velocity greater than zero due to noise. These values for T1 and T2 are based on trial and error learning and are chosen to make sure that displacements due to motion can be detected while at the same rejecting any displacements due to noise. T3 in the ZCR is used as another threshold to detect the zero points, which provides additional information for zero velocity interval detection. It is required in order to ensure accuracy in the detection of the zero velocity intervals. Additionally, these thresholds are also used in detecting the start and end point of the individual elements of the segment movement. Thus, the time taken to perform actions such as the placing of individual pegs in the nine-hole peg test and the overall time to carry out a test can be automatically measured.

**Figure 6:**
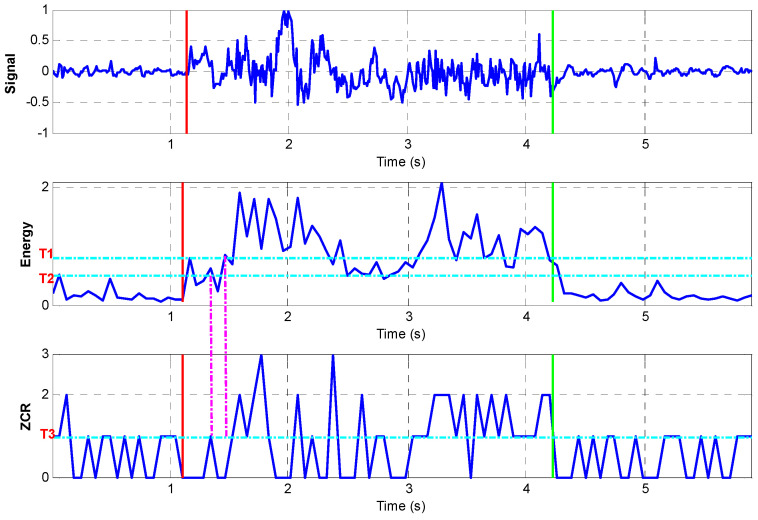
Double threshold method for zero interval detection.

**Figure 7:**
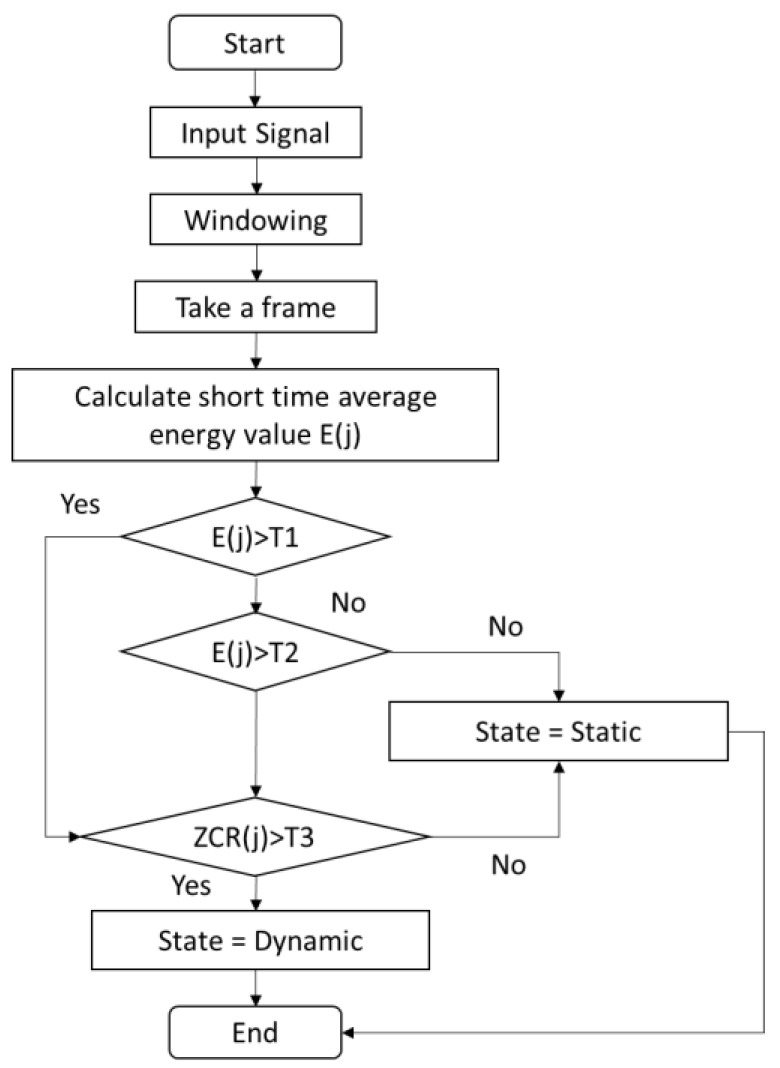
Flow-chart of double threshold method.

**Figure 8:**
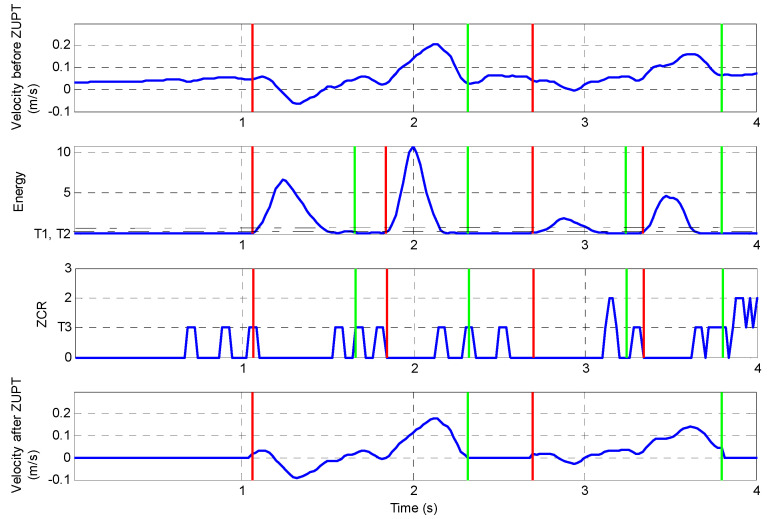
Double threshold method for zero interval detection in nine-hole peg test.

When applying the signal to Equations (5) and (6), the energy and ZCR are calculated. An example of the application of this process is shown in Figure 6 for the first two peg placements in the nine-hole peg test. The details of the process of double threshold method are illustrated in Figure 7. The red line and green line in Figure 8 represent the start and end of each peg insertion movement, respectively. Since one of the features is that the peaks in the velocity signal correspond to the peaks in the gyro energy, the start and end points in the velocity signal can be figured out. This algorithm will start from the first frame (according to the short-term energy), the comparison will be made between the low threshold value T2 and the short time energy. If the first point N1 exceeds the value T2 but the next point after N1 does not exceed value T2, this point cannot be treated as start point. If point N1 exceeds the high threshold value T1, N1 is the first start point. The same method is applied for detecting the end-point N2 as well. Now that the zero velocity events can be detected, the ZUPT algorithms can be applied to the measurements from the nine-hole peg test where there will be zero velocity at the point of peg pick up.

### 3.2. Wavelet Analysis

The basis of the wavelet analysis is to decompose a signal (a time-series) into different scale components using wavelets. Fourier analysis deals with the reconstruction of the signal by sinusoidal components whereas the wavelet analysis does this in terms of wavelets. Wavelet analysis can decompose a signal into the optimal approximation (low-frequency part) and high-frequency part with detailed information [49]. Multiple-level decomposition is used. After decomposition, reconstruction of the signal is the process of assembling all the frequency components except for the low-frequency part.

The wavelet transforms the signal s(t) with respect to the wavelet function ψ(t) as defined in Equation (7):

(7) $$S\left(b,a\right)=\frac{1}{\sqrt{a}}\int_{-\infty }^{\infty }\psi^{\prime }\left(\frac{t-b}{a}\right)s\left(t\right)dt$$

where the wavelet function is: $\psi_{a,b}\left(t\right)=\frac{1}{\sqrt{a}}\psi \left(\frac{t-b}{a}\right)$.

The original signal s(t) can be reconstructed by the following Equation (8).

(8) $$s\left(t\right)=\frac{1}{c_{\psi }}\int_{-\infty }^{\infty }\int_{-\infty }^{\infty }S\left(b,a\right)\psi_{a,b}\left(t\right)\frac{dadb}{a^{2}}$$

**Figure 9:**
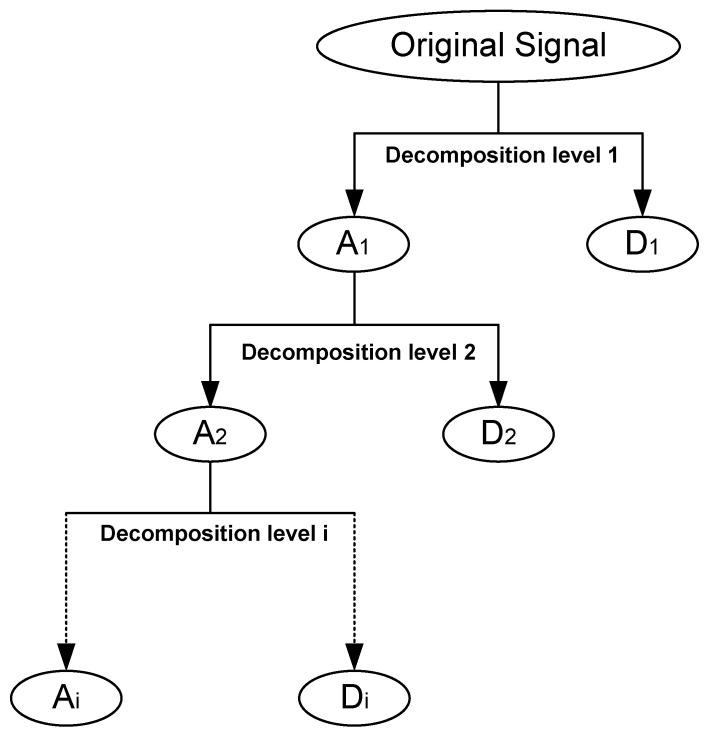
Wavelet analysis decomposition level.

The signal is initially decomposed into two parts—approximation coefficient (A_1_) and detail coefficient (D_1_) and then A_1_ is decomposed into approximation coefficient (A_2_) and detail coefficient (D_2_) at the second decomposition level. A wavelet decomposition tree is presented in Figure 9.

**Figure 10:**
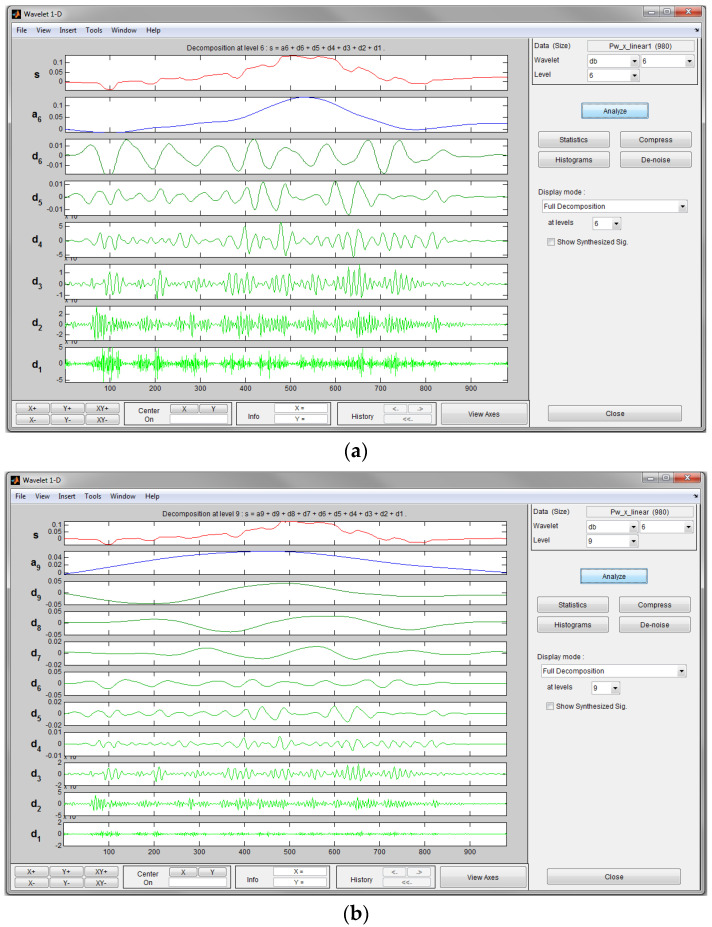
Wavelet analysis decomposition level. (**a**) Wavelet ‘db6’ level 6. (**b**) Wavelet ‘db6’ level 9.

The MATLAB Wavelet toolbox is used to present in a more direct way as shown in Figure 10. This example is the wavelet decomposition of the position tracking data after integrating the acceleration. The wavelet ‘db6’ is selected because its shape is similar to the analysed signal. This given example has chosen wavelet ‘db6’, and the decomposition level chosen is 6. Another example chooses wavelet ‘db6’, and the decomposition level chosen is 9. In this case, the wavelet type (‘db6’) and decomposition level (‘9’) are based on experience and a trial-and-error process.

### 3.3. High-Pass (HP) Filter

**Figure 11:**
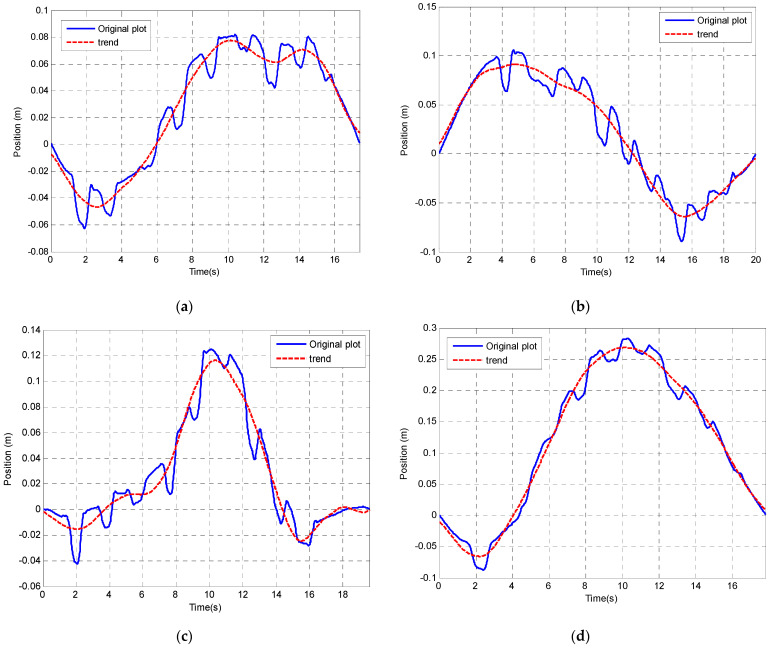
Low frequency offset variation after double integration (red plot is the low frequency trend). (**a**) Experiment Test 1. (**b**) Experiment Test 2. (**c**) Experiment Test 3. (**d**) Experiment Test 4.

High pass filtering has been shown to be effective in digitally removing the DC component offset. The random walk presented in the drift is very similar to a DC component offset. Therefore, an alternative technique of removing offsets and low frequency changes in offset is to apply a HP filter. Figure 11 shows that the low frequency fluctuations after double integration are about 0.05 Hz.

The source of this error may come from the orientation estimation algorithm since the orientation has been used in calculating the linear acceleration in order to remove the component due to gravity. The orientation estimation algorithm uses all acceleration, gyroscope, magnetometer outputs based on sensor fusion. Considering the characteristics of the variations in offset, MATLAB (Filter Designer) was used to create an HP filter with a cut-off frequency of 0.3 Hz to provide an initial evaluation of the effect of applying the filter.

## 4. Results and Discussion

### 4.1. Results Using ZUPT

**Figure 12:**
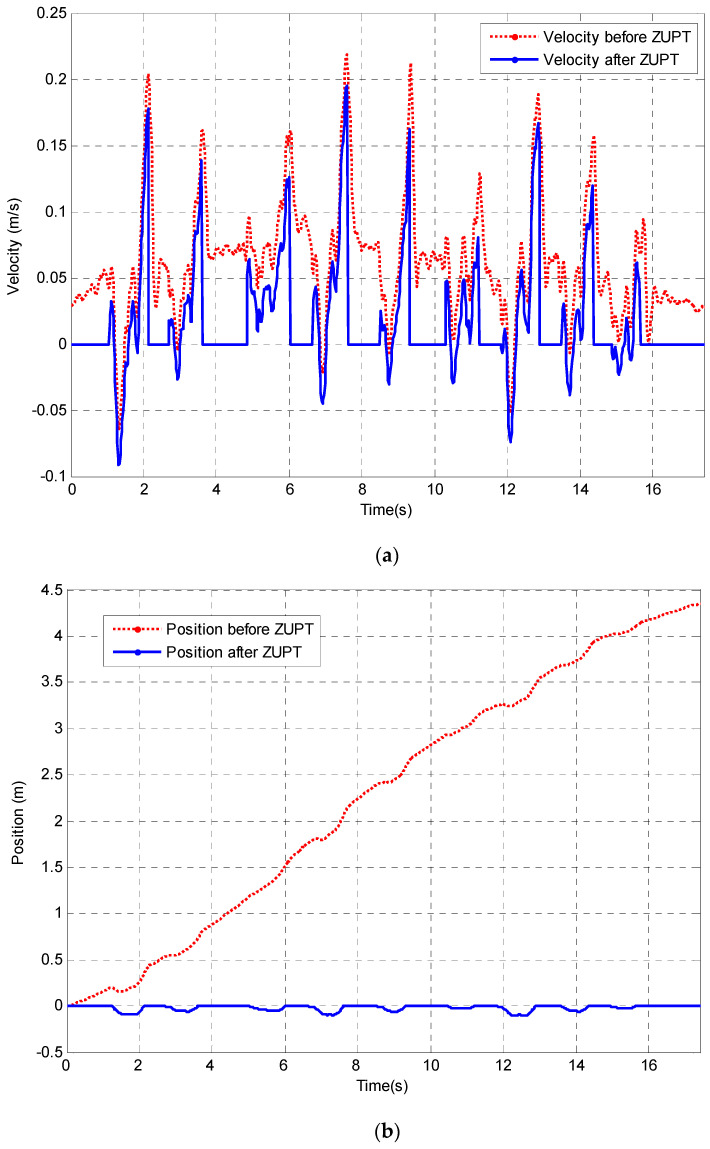

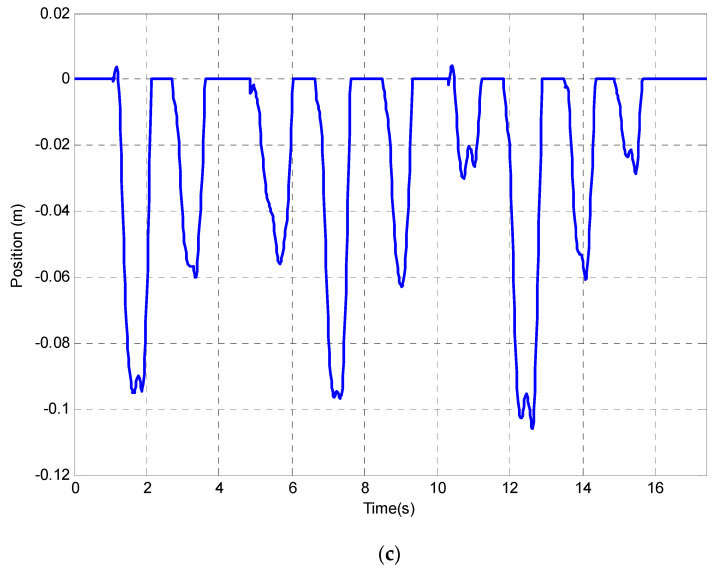
Comparison of hand position tracking by using DR (with and without ZUPT correction). (**a**) Velocity integrated from acceleration before and after ZUPT. (**b**) Position tracking before and after ZUPT. (**c**) Position tracking after ZUPT.

In order to investigate the feasibility of using only a single sensor for tracking hand position, the DR method is applied to the NHPT with data from volunteers. As discussed in the previous sections, the presence of errors in the estimate of offsets in the acceleration and the effect of the presence of noise must be minimised. One methodology is to apply the ZUPT algorithm and an offset correction whenever the velocity is known to be zero. In the NHPT there will be a small pause—zero velocity, at peg pick up and at peg insertion into the hole. The velocity and position plot before and after the ZUPT correction for a healthy volunteer’s movement in the z-direction (horizontal direction with respect to the test board) in the trunk reference frame is shown in Figure 12a,b, respectively. Additionally, the corrected position tracking result is presented in Figure 12c.

As can be seen in Figure 12, ZUPT has set position to zero during the detected zero velocity intervals of peg pick-up. This means that any movement during the estimated zero intervals has been ignored and will add an error to the estimate of position. It was observed that one current disadvantage of applying the ZUPT is that there is a loss of data which made it necessary to scale the ZUPT position data to that obtained from the kinematic model for presentation in Figure 12. One cause of this scale factor is thought to be the data loss in the interval where the velocity is assumed to be zero.

When the DR algorithm is used, the application of the ‘ZUPT’ correction can reduce errors introduced through the double integration of acceleration from several meters to 0.8% of the total movement distance. Additionally, it should be noted that the algorithm of the double threshold method can also be used in the automatic measurement of the timing of segment movements such as individual peg pick-up and placement. This will provide the clinician with additional data for the assessment of patient performance.

### 4.2. Results Using Wavelet Analysis

**Figure 13:**
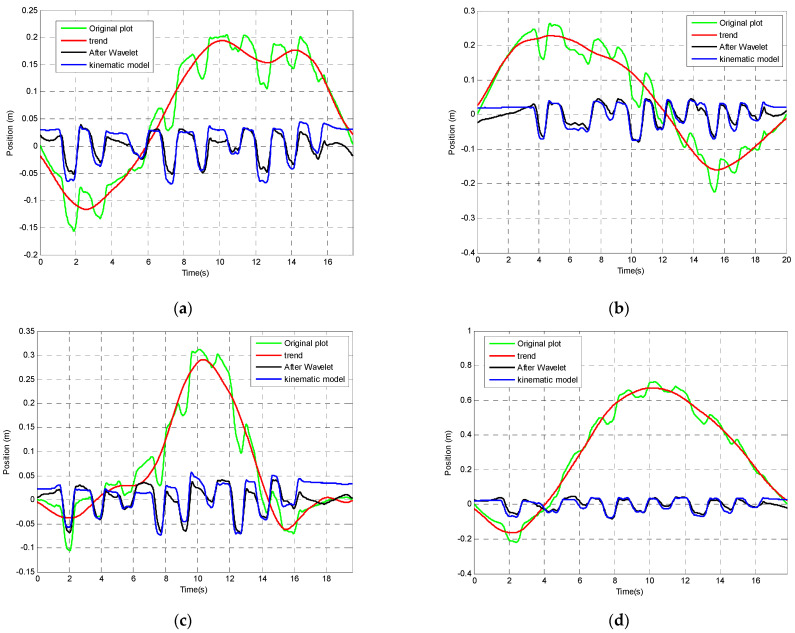
Drift correction for position using wavelet analysis. (**a**) Experiment Test 1. (**b**) Experiment Test 2. (**c**) Experiment Test 3. (**d**) Experiment Test 4.The result after applying the correction using wavelet analysis has been compared with the kinematic modelling tracking result which has been treated as a more accurate position tracking method (See Figure 14).

The correlation coefficient between the corrected data using the wavelet correction and the referenced kinematic modelling result is used as the criteria of evaluating the effectiveness of the chosen wavelet decomposition. The results indicate that wavelet ‘db6’ decomposed in level 9 (correlation coefficient is 0.88) is better than that of the same wavelet decomposed in level 6 (correlation coefficient is 0.36). Therefore, in the following drift correction analysis, wavelet ‘db6’ is chosen and the position signal has been de-composed in level 9. Components D1, D2, D3, D4, D5, D6 and D7 have been used to reconstruct the position. Components D8, D9 and A9 are used to construct the drift trend. The original position signal plot, drift trend estimated by the wavelet, corrected signal after using wavelet as well as kinematic model position estimation result have been shown in Figure 13.

**Figure 14:**
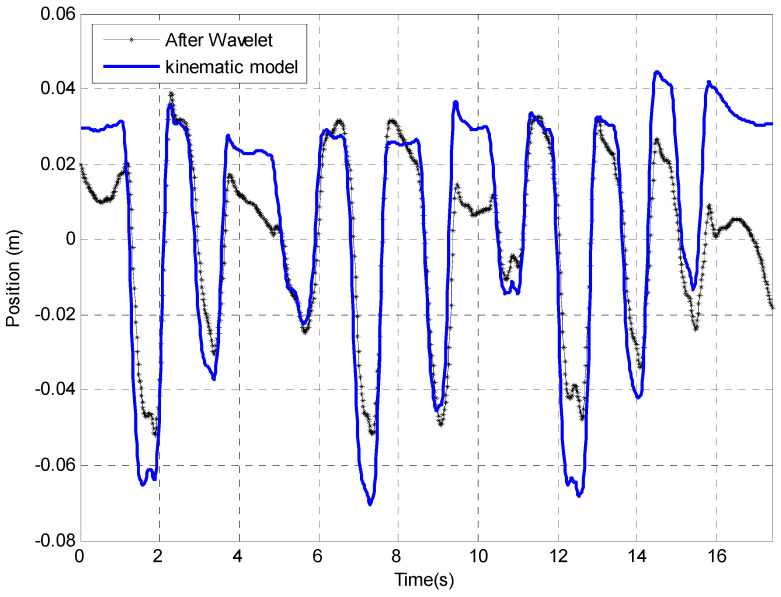
Drift removed by wavelet compared with the kinematic model.

In the above Figure 14, wavelet ‘db6’ has been used in the plot and has been decom-posed in level 9. Though the drift trend has been greatly reduced, the error in each peg movement is still significant, e.g., the maximum error is about 2 cm, which may indicate that this method may not be acceptable for low-frequency error correction especially for this NHPT.

### 4.3. Results Using HP Filter

**Figure 15:**
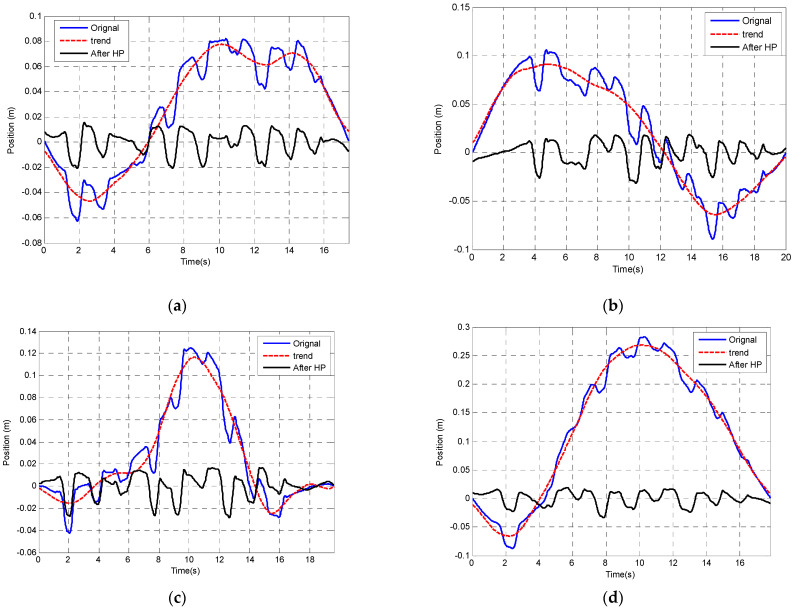
Drift correction result after applying the 0.03 Hz HP filter. (**a**) Experiment Test 1. (**b**) Experiment Test 2. (**c**) Experiment Test 3. (**d**) Experiment Test 4.

An alternative technique of removing offsets and low-frequency changes in offset is to apply a HP filter. Figure 15 shows that the low-frequency fluctuations after double integration are about 0.05 Hz.

The source of this error may come from the orientation estimation algorithm since the orientation has been used in calculating the linear acceleration in order to remove gravity. The orientation estimation algorithm uses all acceleration, gyroscope, and magnetometer outputs based on sensor fusion. Considering the characteristics of the variations in offset, MATLAB (Filter Designer) was used to create a HP filter with a cut-off frequency of 0.3 Hz to provide an initial evaluation of the effect of applying the filter.

The result after applying the correction has also been compared with the Kinematic modelling tracking result which is assumed to be the most accurate position-tracking method (Figure 15). The trend has been marked by the red plot. The blue plot is the original plot and the black plot is corrected after the HP filter.

**Figure 16:**
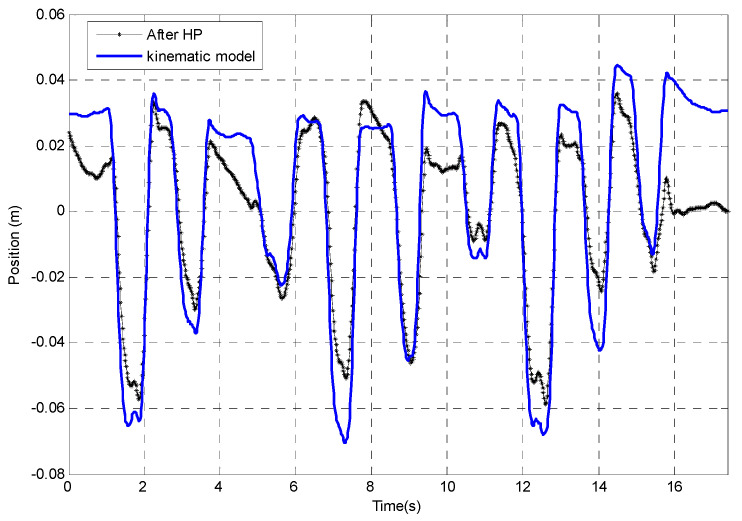
Drift removal by HP filter compared with kinematic model tracking result.

Although the position tracking after using the HP filter correction seems to have improved, it still deviates from the position computation by the kinematic model, especially at the beginning and the end of the plot (Figure 16).

### 4.4. Comparisons between Different Drifts Correction Methods

**Figure 17:**
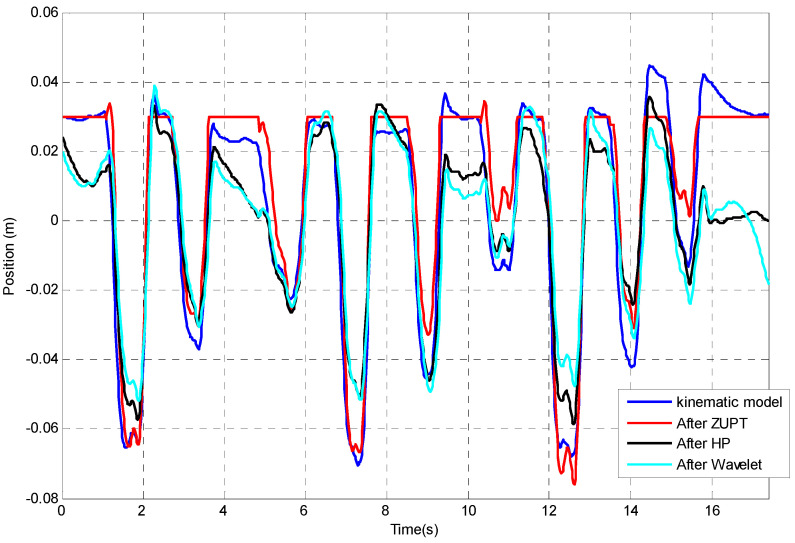
Comparison between the drift correction methods.

A comparison of the different drift correction methods is shown in Figure 17. The spearman correlation coefficient has been calculated in comparing the different drifts correction method, in which the plots after different correction methods are compared with the kinematic modelling. A perfect correlation is close to 1 or −1. Table 1 below shows the correlation coefficient. Besides, by using the kinematic modelling result as the reference result, the mean and standard deviation of the error of using different correction techniques have been presented in Table 2.

From the results of these two tables, ZUPT, as might be expected from a visual inspection of the plots, gives the best correction method. Therefore, the ZUPT is recommended as the method to correct the integration error when there are regular zero velocity intervals. HP can also be used in cases where there are no zero intervals available.

The experiments performed in this work are only on the healthy volunteer. ZUPT showed the best performance as there are clear zero velocities during the phases in NHPT for a healthy subject. For patients who are under neurological rehabilitation, it is still possible to use ZUPT but different thresholds will need to be chosen on the consideration that the patients may have jerky movements or use wrist and forearm to compensate for the hand movement. Furthermore, HP and Wavelet methods are not affected by the zero velocity requirements and can still be used to correct the drifts from the dead reckoning method. Therefore, in future work, we would like to explore the feasibility of applying ZUPT to neurological patients.

## 5. Conclusions

This paper investigates the possibility of using only one inertial sensor to monitor upper limb motion by utilising the dead reckoning method. The effect of errors in the estimate of offsets, drifts in those offsets and the presence of white noise present significant challenges to position estimation for measurement periods of more than a few seconds, e.g., 0.01 m/s^2^ error in the estimate of acceleration can result in a 9 m error in position estimate after 30 s. Therefore, three drift correction methods were assessed. These are ZUPT, HP filter, and wavelet analysis. Initial analysis indicates that when there are regular zero velocity intervals present the ZUPT is the most effective drift correction method. The use of an HP is recommended for those measurement regimes where readily identifiable zero velocity intervals are not present in the data.

An additional benefit of the ZUPT is that the zero-interval detection method—the double threshold method can also be used in the automatic measurement of timing intervals for specific segment movements during an assessment. This will provide the clinician with additional information not available through observation and the use of a stopwatch.

## Figures and Tables

**Table 1:** Coefficient comparison between the drift removal methods.

Correlation Coefficient	ZUPT	HP	Wavelet
Kinematic model	0.97	0.92	0.88

**Table 2:** Mean and standard deviation of error after different drifts correction methods.

(cm)	ZUPT	HP	Wavelet
Mean	0.48	0.49	0.50
STD	0.86	1.43	1.65

## Data Availability

The data presented in this study are available on reasonable request from the corresponding author.
